# Impact of Malocclusions on Periodontopathogenic Bacterial Load and Progression of Periodontal Disease: A Quantitative Analysis

**DOI:** 10.3390/microorganisms12081553

**Published:** 2024-07-29

**Authors:** Ştefan-Dimitrie Albu, Ioana Suciu, Cristina-Crenguţa Albu, Anca-Oana Dragomirescu, Ecaterina Ionescu

**Affiliations:** 1 Department of Periodontology, Faculty of Dentistry, “Carol Davila” University of Medicine and Pharmacy, 37 Dionisie Lupu Street, 020021 Bucharest, Romania; stefan-dimitrie.albu@drd.umfcd.ro; 2 Department of Endodontics, Faculty of Dentistry, “Carol Davila” University of Medicine and Pharmacy, 37 Dionisie Lupu Street, 020021 Bucharest, Romania; ioana.suciu@umfcd.ro; 3 Department of Genetics, Faculty of Dentistry, “Carol Davila” University of Medicine and Pharmacy, 37 Dionisie Lupu Street, 020021 Bucharest, Romania; 4 Department of Orthodontics and Dentofacial Orthopaedics, Faculty of Dentistry, “Carol Davila” University of Medicine and Pharmacy, 37 Dionisie Lupu Street, 020021 Bucharest, Romania; anca.dragomirescu@umfcd.ro (A.-O.D.); ecaterina.ionescu@umfcd.ro (E.I.)

**Keywords:** malocclusions, periodontal disease, periodontopathogenic bacteria, bacterial quantification, real-time PCR

## Abstract

Background: (1) Periodontal disease (PD) is a globally prevalent chronic inflammatory condition, exacerbated by the dysbiosis of the oral microbiota. This study aims to evaluate the bacterial load of specific periodontopathogenic bacteria in patients with malocclusions (MAL) compared to those without. (2) Methods: Conducted at the “Carol Davila” University of Medicine and Pharmacy, Bucharest, Romania, this pilot study involved two groups: patients with MAL and PD, and patients with PD but without MAL. We included 20 patients: 10 with MAL (9 with crowding and 1 with an open bite) and 10 without MAL. Gingival crevicular fluid was collected for bacterial DNA extraction and quantified bacterial load using real-time PCR, focusing on 12 periodontopathogenic bacteria across different complexity classes. (3) Results: The study identified significantly higher concentrations of *Treponema denticola* (*p* = 0.023, median = 4.32, IQR = 2.76–5.53 vs. median = 1.93, IQR = 0–3.19), *Tannerella forsythia* (*p* = 0.020, mean = 6.04 ± 0.72 vs. mean = 4.4 ± 1.89) and *Porphyromonas gingivalis* (*p* = 0.002, median = 5.64, IQR = 4.94–5.98 vs. median = 2.48, IQR = 0–4.05) in patients with MAL compared to those without. This suggests that MAL contributes to an environment conducive to the proliferation of specific pathogens, potentially accelerating PD progression. Additionally, *Eikenella corrodens* (*p* = 0.040, mean = 4.55 ± 1.02 vs. mean = 3.23 ± 1.56), *Campylobacter rectus* (*p* < 0.001, mean = 4.2 ± 0.56 vs. mean = 1.8 ± 1.51), *Prevotella intermedia* (*p* = 0.043, median = 5.04, IQR = 0–5.49 vs. median = 0, IQR = 0–3.39), *Capnocytophaga sputigena* (*p* = 0.011, median = 5.91, IQR = 5.47–6.17 vs. median = 4.63, IQR = 3.83–5.64), and *Capnocytophaga gingivalis* (*p* = 0.007, median = 5.87, IQR = 5.34–6.03 vs. median = 4.4, IQR = 3.5–5.71) also showed elevated concentrations, indicating the broad impacts of MAL on oral microbial profiles. (4) Conclusions: The findings demonstrate a significant relationship between MAL and increased bacterial loads, underscoring the need for its integration in managing PD. Future research should expand demographic diversity and employ longitudinal designs to better understand the causative mechanisms at play.

## 1. Introduction

Periodontal disease (PD) is a multifactorial chronic inflammatory disease affecting both soft and hard periodontal structures [1]. According to the World Health Organization (WHO), PD is the second most common adult condition after dental caries [2]. In addition, findings from the Global Burden of Disease study emphasize that severe forms of PD are the 11th most prevalent disease worldwide, affecting between 20% and 50% of the global population [3].

Currently, in relation to the different clinical forms of PD, gingivitis and mild forms of periodontitis affect 40–60% of the global population, while 10–15% of the population have severe forms of periodontitis [4], with the latter representing a major cause of tooth loss, which results in negative consequences on the patients’ quality of life [2,5]. 

PD is characterized by the progressive destruction of the soft and hard tissues of the periodontal complex. This destructive process is initiated and sustained through interactions between dysbiotic oral microbial communities and aberrant host immune responses against the resident bacterial biofilm in gingival and periodontal tissues [6].

The oral cavity, due to its unique anatomical structures, is one of the most heavily colonized areas of the human body, hosting an astonishing variety of microorganisms, including bacteria, archaea, fungi, mycoplasmas, protozoa, and viruses. Under normal conditions, these microorganisms coexist in complete harmony, forming a balanced ecosystem. [7]. 

The entirety of microorganisms in the oral cavity, their genetic information, and the oral environment in which they interact is called the oral microbiome, while the entirety of living microorganisms that make up the oral microbiome is called the oral microbiota [7]. “Oral microbiome”, “oral microflora”, or “oral microbiota” are the terms most frequently used to describe the microbial community residing in the human oral cavity [8,9]. The oral microbiota consists of three major entities: (1) the oral bacteriome—the bacterial component; (2) the oral mycobiome—the fungal component; and (3) the oral virome or oral virobiome—the viral component [10].

Oral bacteria, as essential constituents of the human oral bacteriome, can be systematically classified on several criteria: (1) Gram-positive and Gram-negative bacteria, distinguished by their Gram staining results; (2) cocci, bacilli, and spirochetes, classified according to their morphological characteristics; and (3) aerobic, facultatively anaerobic, micro-aerophilic, and strictly anaerobic organisms, categorized by their oxygen tolerance [11].

Next Generation Sequencing (NGS) technology, used to investigate the structure, function, and diversity of the human oral microbiome, has clearly demonstrated that the oral microbiome is unique and specific to each individual. In addition, the composition of oral microbiota in healthy individuals differs remarkably from person to person [10].

In conditions of periodontal health, the oral microbiome is a well-balanced and dynamic ecosystem. However, as the resident oral microbiota becomes dysbiotic, periodontal pathogens become more numerous, exacerbating inflammatory responses, which in turn induce tissue destruction through a positive feedback mechanism involving proteolysis, inflammation, and the multiplication of periodontal pathogens [6,10,12].

As a result, once the dysbiosis of the oral bacteriome is established, it creates an imbalance that affects the relative abundance of microbial species and genera, significantly impacting the host and inducing the development of PD in susceptible patients [6,10]. Essentially, in a dysbiotic oral microbiome, the diversity and relative proportions of species or taxa in the oral microbiota are disrupted [10,12].

Although the constitutive diversity of the oral bacteriome remains largely unexplained, local individual promoting factors, such as MAL, caries, edentulism, parafunctions, vicious habits, together with systemic individual promoting factors, environmental factors, host genetic specificity, and early microbial exposure play a significant role in shaping the composition of the oral microbiota and exacerbating the severity of PD [10,13]. Identifying and elucidating the mechanisms of action of these factors may provide valuable insights into the holistic prevention and efficient management of PD.

MAL is a dental condition characterized by the misalignment of teeth and jaws [14]. Its etiology is multifactorial, encompassing genetic, environmental, and developmental factors [15]. The two types of MAL included in this study are teeth crowding and open bite. Teeth crowding occurs when there is insufficient space in the dental arch for all teeth to align properly, resulting in overlapping or crooked teeth [14]. An open bite is characterized by a vertical gap between the upper and lower teeth when the mouth is closed, preventing proper contact between them [16]. The ways in which MAL are already known to contribute to PD include preventing effective oral hygiene, disrupting salivary flow, retaining food particles, and altering the distribution of occlusal forces [17]. These factors collectively create an environment conducive to the proliferation of periodontal pathogens, exacerbating the progression of PD [18].

The aim of this study was to evaluate and statistically compare the bacterial loads of 12 different species of periodontopathogenic bacteria between two groups of patients: (1) patients with malocclusions (MAL) and PD; and (2) patients with PD but without MAL. The 12 species of periodontopathogenic bacteria studied were the following:

- Purple complex bacteria: *Aggregatibacter actinomycetemcomitans*.
- Red complex bacteria: *Porphyromonas gingivalis*, *Treponema denticola*, and *Tannerella forsythia*.
- Orange complex bacteria: *Eikenella corrodens*, *Campylobacter rectus*, *Prevotella intermedia*, *Fusobacterium nucleatum*, and *Prevotella nigrescens*.
- Green complex bacteria: *Capnocytophaga ochracea*, *Capnocytophaga sputigena*, and *Capnocytophaga gingivalis*.

The purple complex bacteria, such as *Aggregatibacter actinomycetemcomitans*, are early colonizers often associated with aggressive forms of PD [19]. The red complex bacteria, including *Porphyromonas gingivalis, Treponema denticola*, and *Tannerella forsythia*, are highly pathogenic and strongly linked to the severity and progression of PD [20,21]. The orange complex bacteria, such as *Eikenella corrodens, Campylobacter rectus, Prevotella intermedia, Fusobacterium nucleatum*, and *Prevotella nigrescens*, play a crucial role in the maturation of dental plaque and facilitate the colonization of red complex bacteria [21,22]. The green complex bacteria, including *Capnocytophaga ochracea, Capnocytophaga sputigena,* and *Capnocytophaga gingivalis*, are less pathogenic but contribute to the overall microbial ecosystem and influence disease progression [23].

## 2. Materials and Methods

### 2.1. Study Design and Participants

The present research is a pilot study conducted between November 2022 and April 2024 at the Clinical Department of Periodontology at the Faculty of Dentistry, “Carol Davila” University of Medicine and Pharmacy, Bucharest, Romania.

The study protocol was approved by The Scientific Research Ethics Commission of the “Carol Davila” University of Medicine and Pharmacy, Bucharest, Romania (Protocol number: 27652/02.02.2024), and was guided in accordance with the Declaration of Helsinki of 1975. Informed consent was obtained from all participating patients.

All participants were divided into two study groups.

#### 2.1.1. Study Group 1: Patients with PD and MAL

Inclusion criteria were as follows:

- Patients with PD and MAL represented by crowding and open bite, who presented to the Clinical Department of Periodontology at the Faculty of Dentistry, “Carol Davila” University of Medicine and Pharmacy, Bucharest, Romania, for consultation and specialized treatment during the selected period.
- Cooperative Caucasian patients of both sexes who agreed to be included in the study.
- Patients aged between 30 and 40 years.
- Patients in generally good health.
- Patients with at least 24 teeth present.
- Patients who have not been treated for PD in the last year (such as descaling or brushing).
- Patients who have not undergone orthodontic treatment.
- Patients without dental extractions, except for three molars.
- Patients without dental implants or different dental prosthetic works.
- Patients who have not received antibiotic treatment in the last six months.

Exclusion criteria:

- Patients not meeting the inclusion criteria.
- Pregnant women.
- Patients with chronic debilitating diseases (including diabetes mellitus, neoplasms, systemic diseases, autoimmune diseases, cirrhosis, acute infectious diseases).
- Patients under chronic treatment with anti-inflammatory drugs and corticotherapy.

#### 2.1.2. Study Group 2: Patients with PD without MAL

Inclusion criteria were as follows:

- Patients with PD but without MAL, who presented to the Clinical Department of Periodontology at the Faculty of Dentistry, “Carol Davila” University of Medicine and Pharmacy, Bucharest, Romania, for consultation and specialized treatment during the selected period.
- Cooperative Caucasian patients of both sexes who agreed to be included in the study.
- Patients aged between 30 and 40 years.
- Patients in generally good health.
- Patients with at least 24 teeth present.
- Patients who have not been treated for PD in the last year (such as descaling or brushing).
- Patients who have not undergone orthodontic treatment.
- Patients without dental extractions, except for three molars.
- Patients without dental implants or different dental prosthetic works.
- Patients who have not received antibiotic treatment in the last six months.

Exclusion criteria were as follows:

- Patients not meeting the inclusion criteria.
- Pregnant women.
- Patients with chronic debilitating diseases (including diabetes mellitus, neoplasms, systemic diseases, autoimmune diseases, cirrhosis, acute infectious diseases).
- Patients under chronic treatment with anti-inflammatory drugs and corticotherapy.

#### 2.1.3. Patient Selection Process

During the study period, a total of 1960 patients presented to the Clinical Department of Periodontology at the Faculty of Dentistry, “Carol Davila” University of Medicine and Pharmacy, Bucharest, Romania. Based on the selection criteria outlined in our study protocol, we initially identified 28 potential participants. Of these, 20 patients met the inclusion criteria and were ultimately selected for the study: 10 patients with PD and MAL, and 10 patients with PD without MAL. The 12 remaining patients were excluded for various reasons, including the onset of acute diseases requiring antibiotic treatment (4 cases) or anti-inflammatory treatment (2 cases), pregnancy (1 case), and lack of cooperation (1 case).

### 2.2. Methods

#### 2.2.1. Medical Records

Demographic data (age, sex, education level, family history, smoking habits) along with clinical and diagnostic information were systematically gathered from the patients’ periodontal records.

#### 2.2.2. Collection of the Gingival Crevicular Fluid

Gingival crevicular fluid (GCF) samples from periodontal pockets were collected in the clinic by the dentist using a special sterile collection kit consisting of five paper tips and one transfer tube.

GCF samples were collected by swabbing with the sterile paper tips, each inserted into a different periodontal pocket for approximately 10 s. Afterwards, the paper tips were transferred into the sterile tube and sent to the laboratory for DNA extraction and bacterial load quantification.

#### 2.2.3. Bacterial DNA Extraction

The paper tips were kept overnight in 1 mL of 1–1.5× PBS (phosphate buffered saline) solution at 4–8 °C. An incubation of approximately 4 h at 37–40 °C with shaking was added for maximum extraction. The liquid from each tube was completely transferred into a new 1.5 mL tube and centrifuged for 10 min at 14,000 RPM. The sediment, together with approximately 100–150 µL of PBS, was subsequently processed with a gDNA extraction kit (Favorgen Biotech Corp., Ping-Tung, Taiwan; Qiagen, Hilden, Germany) according to the manufacturer’s instructions. The biological material was subjected to lysis with a specified volume of buffer for approximately 10 min at room temperature and then treated with a specific volume of 100% ethanol and loaded into columns with nucleic acid retention silica. After a first centrifugation (3 min at 14,000 RPM), the columns were successively loaded with washing solutions specific to the kit and re-centrifuged. A final dry centrifugation ensured the complete elimination of traces of the solution from the columns. Finally, the columns were hydrated with 80 µL of water and centrifuged into new tubes to elute the total nucleic acids from the sample.

#### 2.2.4. Quantification by Real-Time PCR (qRT-PCR)

Bacterial quantification was performed using a Bio-Rad CFX-96 Real-Time System with Bio-Rad CFX Maestro 2.0 software (Bio-Rad Laboratories, Inc., Hercules, CA, USA). A reaction mix specific to each bacterial species was loaded into a 96-well PCR plate in a predetermined order. Each well contained 14 μL of Qiagen RT² SYBR Green ROX qPCR Mastermix (Qiagen, Hilden, Germany), 0.75 μL of each forward and reverse primer, and 4.1 μL of sample, totaling approximately 20 μL per reaction. The primers were sourced from the literature [24,25,26], tested, validated, and updated according to the latest Ensembl data (2023).

The amplification reaction included an initial DNA denaturation at 95 °C for 5 min, followed by 40 cycles of 95 °C for 10 s, 60 °C for 5 s, and 72 °C for 25 s, with fluorescence recording. High-resolution melting (HRM) analysis was performed to detect species-specific dissociation temperatures. Relative quantification was achieved by comparing results to a standard curve generated from genomic DNA of Porphyromonas gingivalis, titrated to a density of 6.0 × 10^6^ colony-forming units (CFUs). The protocol was adapted from Cosac et al. (2017) for use with the Bio-Rad CFX-96 Real-Time System [27].

#### 2.2.5. Principle of the Detection Method

The ParoIDENT genetic test is based on the independent amplification of the genetic material specific to each of the 12 bacterial strains through RT-PCR reactions (polymerization reaction monitored in real time) and the confirmation of its specificity is achieved through the HRM analysis of the amplified fragments of the bacterial genetic material.

RT-PCR amplification step. The results were evaluated based on the PCR cycle threshold (Ct), where the fluorescent signal of SYBR Green attached to double-stranded DNA amplicons exceeds the background noise, typically 1000 RFU (relative fluorescence units). The Ct value was noted for each reaction, and only reactions with Ct < 36 were included to avoid false positives. The RT-PCR method with SYBR Green highlights the fluorophore’s affinity for double-stranded DNA. Specificity for bacterial targets was confirmed in the HRM stage.

High-resolution melting stage. Specificity of the amplification was confirmed post-PCR by gradually denaturing the amplicons and observing the decrease in the SYBR Green fluorescent signal as it dissociated from single-stranded DNA. The resulting dissociation curve, which features three segments (pre-dissociation, active dissociation, and post-dissociation), helped confirm the specificity of the amplified genetic material.

Detection of “dissociation peaks”—peak detection. Within the fast dissociation region, approximately 50% of the double-stranded DNA becomes single-stranded, marking the maximum rate of fluorescence signal decrease (melting temperature, Tm). An algorithm calculates the dissociation rate as a function of temperature to represent these data as a peak corresponding to Tm. Each tested bacterium has a theoretical Tm based on the species-specific 16S amplicon. Differences in nucleotide structure and amplicon length establish Tm standards and the graphical appearance of the dissociation data.

Quantification of the bacterial load. The absolute concentration of bacteria in periodontal pockets was estimated through a proportional relationship between the fluorescent signal emitted during the RT-PCR reaction and a fluorescent scale specific to the serial dilutions of the anaerobic bacterial culture. The bacterial load was quantified using a scale based on colony-forming units (CFUs). Relative quantification used a standard amplification curve from genomic DNA corresponding to serial dilutions starting from a pure culture of *Porphyromonas gingivalis* at a concentration of 6.0E6 CFU. The Ct for one of the serial dilutions reflected the amount of genetic material extracted and amplified using the same RT-PCR method. Similar scales were obtained for other bacteria (e.g., *Fusobacterium nucleatum*, *Fusobacterium periodonticum*) with statistically identical results.

Practical aspects. Considering the variations in the composition of the genetic material extracted from the samples, the simultaneous processing of multiple samples allows the establishment of Tm ranges and the graphical appearance of the dissociation curves specific to each tested bacterium. The atypical dissociation and Tm curves were specifically excluded from reporting. A final sample list, identical to that on the machine, was used to mark validity and exclude samples with the non-specific amplification and Ct values below 36. Excluded samples were reported as negative, while positive samples were reported with the Ct value obtained from the device.

Evidence reporting. For each patient, the data were transferred from the device to Excel, and the results were expressed numerically and graphically. Values close to 1 × 10^3^ CFU for *Aggregatibacter actinomycetemcomitans*, 1 × 10^4^ CFU for *Porphyromonas gingivalis*, *Treponema denticola*, and *Tannerella forsythia*, and 1 × 10^5^ CFU for *Eikenella corrodens, Campylobacter rectus*, *Prevotella intermedia*, *Fusobacterium nucleatum*, *Fusobacterium periodonticum*, and *Prevotella nigrescens*, and 1 × 10^6^ CFU for *Capnocytophaga ochracea*, *Capnocytophaga sputigena*, and *Capnocytophaga gingivalis* are considered indicative of periodontal pathogenic risk. Ultimately, the interpretation of the results remains at the discretion of the doctor, emphasizing that the evaluation must consider both the bacterial load and spectrum, as well as the evolution of bacterial flora before and after treatment.

#### 2.2.6. Statistical Analysis

All the data from the study were analyzed using IBM SPSS Statistics for Windows, Version 25.0 (IBM Corp., Armonk, NY, USA) and illustrated using Microsoft Office Excel/Word 2021 (Microsoft Corp., Redmond, WA, USA). Quantitative variables were tested for normal distribution using the Shapiro–Wilk Test and were written as averages with standard deviations or medians with interquartile ranges. Qualitative variables were written as counts or percentages and were compared between groups using Fisher’s Exact Test.

Bacterial concentrations were calculated as log10 concentrations in order to facilitate an easier comparison and visualization between groups. Quantitative variables with normal distribution were compared between groups using the Student’s *t*-test or Welch’s *t*-test (according to the equality of variances observed using Levene’s Test) while quantitative variables with non-parametric distribution were tested between groups using the Mann–Whitney U Test.

## 3. Results

Data from Table 1 show the characteristics of the analyzed patients. Ten patients without MAL (50%) and 10 patients with MAL (50%) were included in the study. None of the analyzed characteristics were significantly different between groups (*p* > 0.05), and the results show that the patients had a mean age of 37.35 ± 3.16 years (median = 38 years), most of them being men (70%), with a non-academic level of education (55%), and living in an urban environment (55%). Most of the patients were non-smokers (55%) with a family history of periodontitis (55%). All of the patients had periodontitis, having a stage II (50%) or stage III (40%) periodontitis, and in most of patients, the periodontitis was generalized (85%). Regarding MAL, the majority of patients present crowding (90%).

**Figure 1:**
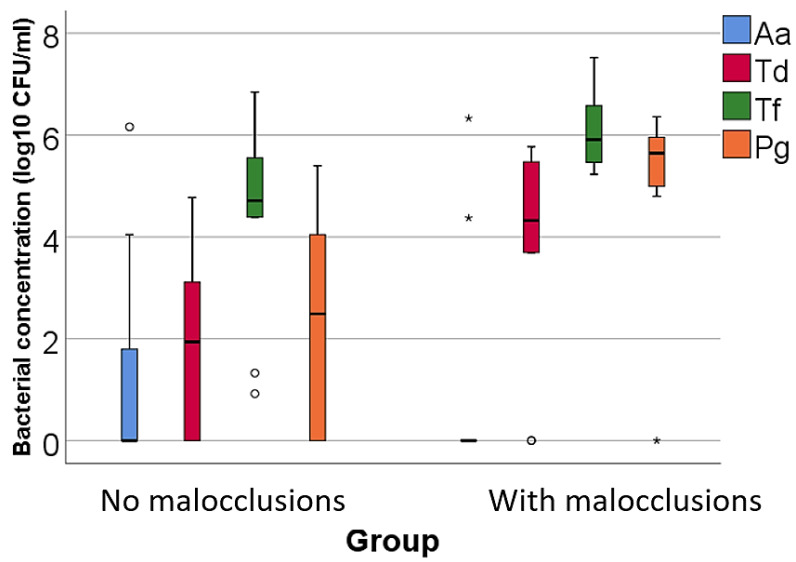
Comparison of isolated high pathogenic bacterial cultures concentrations according to the existence of malocclusions. The figure illustrates that patients with malocclusions have a significantly higher concentration of *Treponema denticola, Tannerella forsythia,* and *Porphyromonas gingivalis* compared to patients without malocclusions. Legend: Aa (*Aggregatibacter actinomycetemcomitans*)—represented by blue bars; Td (*Treponema denticola*)—represented by red bars; Tf (*Tannerella forsythia*)—represented by green bars; Pg (*Porphyromonas gingivalis*)—represented by orange bars; malocclusions (MAL). Box-Plot Components: median (black line inside the box)—indicates the median bacterial concentration for each group; interquartile range (IQR)—the box spans from the 25th percentile (Q1) to the 75th percentile (Q3), representing the middle 50% of the data; whiskers—extend to the smallest and largest values within 1.5 times the IQR from Q1 and Q3, respectively; circles—represent mild outliers, defined as values that fall between 1.5 and 3 times the IQR from the quartiles; asterisks—represent extreme outliers, defined as values that fall beyond three times the IQR from the quartiles. Key observations: Patients with MAL have significantly higher concentrations of Td (*p* = 0.023), Tf (*p* = 0.020) and Pg (*p* = 0.002). These differences are statistically significant.

**Figure 2:**
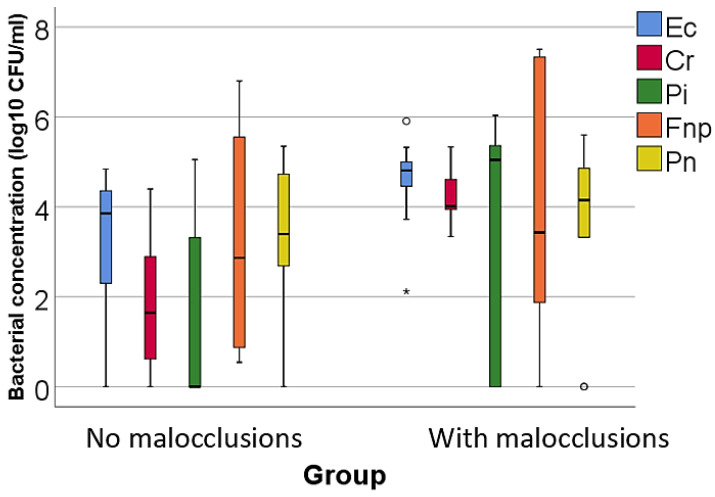
Comparison of isolated moderate pathogenic bacterial cultures concentrations according to the existence of malocclusions. The figure illustrates that patients with malocclusions have significantly higher concentrations of *Eikenella corrodens, Campylobacter rectus*, and *Prevotella intermedia* compared to patients without malocclusions. Legend: Ec (*Eikenella corrodens*)—represented by blue bars; Cr (Campylobacter rectus)—represented by red bars; Pi (*Prevotella intermedia*)—represented by green bars; Fnp (*Fusobacterium nucleatum*): represented by orange bars; Pn (*Prevotella nigrescens*)—represented by yellow bars; malocclusions (MAL). The Box-Plot components for Figure 2 are similar to those described for Figure 1. Box-Plot components: median (black line inside the box)—indicates the median bacterial concentration for each group; interquartile range (IQR)—the box spans from the 25th percentile (Q1) to the 75th percentile (Q3), representing the middle 50% of the data; whiskers—extend to the smallest and largest values within 1.5 times the IQR from Q1 and Q3, respectively; circles—represent mild outliers, defined as values that fall between 1.5 and 3 times the IQR from the quartiles; asterisks—represent extreme outliers, defined as values that fall beyond 3 times the IQR from the quartiles. Key observations: Patients with MAL have significantly higher concentrations of Ec (*p* = 0.040), Cr (*p* < 0.001) and Pi (*p* = 0.043). These differences are statistically significant.

**Figure 3:**
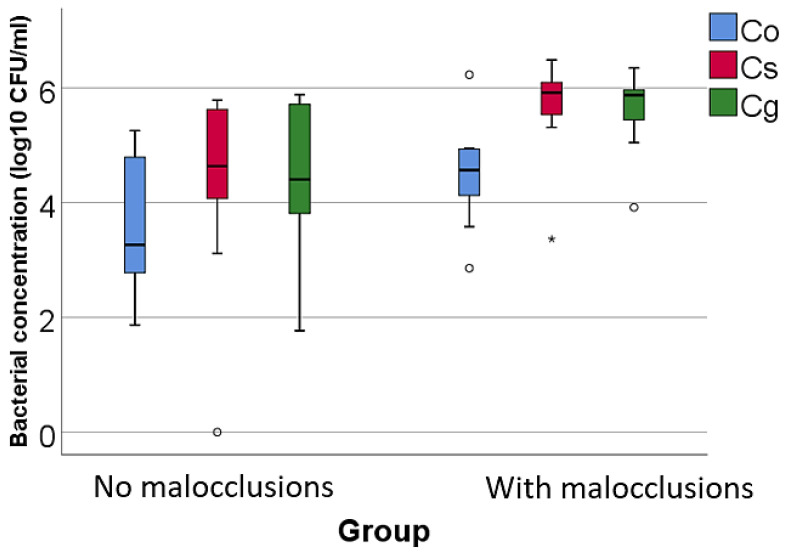
Comparison of the isolated low pathogenic bacterial cultures concentrations according to the existence of malocclusions. The figure illustrates that patients with malocclusions have significantly higher concentrations of *Capnocytophaga sputigena* and *Capnocytophaga gingivalis* compared to patients without MAL. Legend: Co (*Capnocytophaga ochracea*)—represented by blue bars; Cs (*Capnocytophaga sputigena*)—represented by red bars; Cg (*Capnocytophaga gingivalis*)—represented by green bars; malocclusions (MAL). Box-Plot components: median (black line inside the box)—indicates the median bacterial concentration for each group; interquartile range (IQR)—the box spans from the 25th percentile (Q1) to the 75th percentile (Q3), representing the middle 50% of the data; whiskers—extend to the smallest and largest values within 1.5 times the IQR from Q1 and Q3, respectively; circles—represent mild outliers, defined as values that fall between 1.5 and 3 times the IQR from the quartiles.; asterisks—represent extreme outliers, defined as values that fall beyond 3 times the IQR from the quartiles. Key observations: Patients with MAL have significantly higher concentrations of Cs (*p* = 0.011) and Cg (*p* = 0.007). These differences are statistically significant.

Data from Table 2 and Figure 1, Figure 2 and Figure 3 show the comparison of the isolated bacterial cultures concentrations according to the existence of MAL. The results show the following significant differences between groups (*p* < 0.05):

- For high pathogenic bacteria: higher concentrations of *Treponema denticola* (*p* = 0.023, median = 4.32, IQR = 2.76–5.53 vs. median = 1.93, IQR = 0–3.19), *Tannerella forsythia* (*p* = 0.020, mean = 6.04 ± 0.72 vs. mean = 4.4 ± 1.89), and *Porphyromonas gingivalis* (*p* = 0.002, median = 5.64, IQR = 4.94–5.98 vs. median = 2.48, IQR = 0–4.05) in patients with MAL vs. without MAL;
- For moderate pathogenic bacteria: higher concentrations of *Eikenella corrodens* (*p* = 0.040, mean = 4.55 ± 1.02 vs. mean = 3.23 ± 1.56), *Campylobacter rectus* (*p* < 0.001, mean = 4.2 ± 0.56 vs. mean = 1.8 ± 1.51), and *Prevotella intermedia* (*p* = 0.043, median = 5.04, IQR = 0–5.49 vs. median = 0, IQR = 0–3.39) in patients with MAL vs. without MAL;
- For low pathogenic bacteria, higher concentrations of *Capnocytophaga sputigena* (*p* = 0.011, median = 5.91, IQR = 5.47–6.17 vs. median = 4.63, IQR = 3.83–5.64) and *Capnocytophaga gingivalis* (*p* = 0.007, median = 5.87, IQR = 5.34–6.03 vs. median = 4.4, IQR = 3.5–5.71) in patients with MAL vs. without MAL.

**Table 2:** Comparison of isolated bacterial cultures concentrations according to the existence of malocclusions.

Species (log10 CFU/mL)/Group			No Malocclusions	With Malocclusions
High pathogenic	Aa	Mean ± SD	1.33 ± 2.14	1.07 ± 2.3
		Median (IQR)	0 (0–2.35)	0 (0–1.09)
		*p* *	0.631	
	Td	Mean ± SD	1.77 ± 1.73	3.84 ± 2.17
		Median (IQR)	1.93 (0–3.19)	4.32 (2.76–5.53)
		*p* *	0.023	
	Tf	Mean ± SD	4.4 ± 1.89	6.04 ± 0.72
		Median (IQR)	4.71 (3.62–5.65)	5.9 (5.44–6.63)
		*p* **	0.020	
	Pg	Mean ± SD	2.2 ± 2.1	5.06 ± 1.84
		Median (IQR)	2.48 (0–4.05)	5.64 (4.94–5.98)
		*p* *	0.002	
Moderate pathogenic	Ec	Mean ± SD	3.23 ± 1.56	4.55 ± 1.02
		Median (IQR)	3.85 (2.14–4.47)	4.8 (4.27–5.08)
		*p* **	0.040	
	Cr	Mean ± SD	1.8 ± 1.51	4.2 ± 0.56
		Median (IQR)	1.64 (0.46–3.12)	4.01 (3.88–4.6)
		*p* ***	0.001	
	Pi	Mean ± SD	1.52 ± 2.03	3.74 ± 2.61
		Median (IQR)	0 (0–3.39)	5.04 (0–5.49)
		*p* *	0.043	
	Fnp	Mean ± SD	3.19 ± 2.44	4.05 ± 2.95
		Median (IQR)	2.86 (0.8–5.7)	3.43 (1.4–7.33)
		*p* **	0.488	
	Pn	Mean ± SD	3.38 ± 1.53	3.54 ± 1.97
		Median (IQR)	3.39 (2.62–4.79)	4.15 (2.49–4.87)
		*p* *	0.481	
Low pathogenic	Co	Mean ± SD	3.6 ± 1.2	4.49 ± 0.89
		Median (IQR)	3.26 (2.67–4.82)	4.56 (4–4.93)
		*p* **	0.078	
	Cs	Mean ± SD	4.29 ± 1.71	5.66 ± 0.89
		Median (IQR)	4.63 (3.83–5.64)	5.91 (5.47–6.17)
		*p* *	0.011	
	Cg	Mean ± SD	4.33 ± 1.35	5.64 ± 0.71
		Median (IQR)	4.4 (3.5–5.71)	5.87 (5.34–6.03)
		*p* *	0.007	

* Mann–Whitney U Test, ** Student *t*-test, *** Welch *t*-test, N = number of patients, SD = Standard Deviation, IQR = interquartile range, Aa =* Aggregatibacter actinomycetemcomitans,* Td =* Treponema denticola,* Tf =* Tannerella forsythia,* Pg =* Porphyromonas gingivalis,* Ec =* Eikenella corrodens,* Cr =* Campylobacter rectus,* Pi =* Prevotella intermedia,* Fnp =* Fusobacterium nucleatum,* Pn =* Prevotella nigrescens,* Co =* Capnocytophaga ochracea,* Cs =* Capnocytophaga sputigena,* Cg =* Captocytophaga gingivalis*.

## 4. Discussion

The symbiosis between beneficial microorganisms residing in the oral cavity and their pathogenic counterparts is crucial for maintaining oral health. The disruption of this homeostasis and symbiotic balance leads to dysbiosis, which is implicated in the development of PDs, dental caries, oral candidiasis and even systemic diseases [10,28,29].

Although the factors involved in the etiology of PD have been intensively researched, the etiopathogenesis of PD has not yet been fully elucidated [12]. Studies indicate that periodontal health correlates with an oral Gram-positive bacteriome, rich in microorganisms such as *Streptococcus salivarius* and *Rothia mucilaginosa*, while PD is associated with an oral bacteriome governed by Gram-negative anaerobes, rich in microorganisms such as *Porphyromonas gingivalis*, *Tannerella forsythia*, *Treponema denticola*, and *Aggregatibacter actinomycetemcomitans*, which become dominant in the oral community [6,10,28,29,30].

To date, more than 700 bacterial species have been isolated from the oral cavity, of which 400 have been identified in periodontal pockets and 300 in other areas such as the tongue, oral mucosa, and carious lesions [3,28,31]. Some of these oral pathogens are directly involved not only in the initiation of PD and caries but also in the mediation and progression of some systemic diseases such as diabetes, cardiovascular diseases, osteoporosis, atherosclerosis, and tumors [12,29].

Microbiological, genetic and lifestyle susceptibility are hypothesized to be closely related to the induction and severity of PD. The contemporary model of PD pathogenesis underscores the involvement of a diverse bacterial microflora that is more complex than previously thought. According to this model, PD does not solely arise from individual pathogenic microbes but rather emerges from the synergistic interactions among multiple bacterial species and genera, exacerbated by dysbiosis. This dysbiosis disrupts the balanced ecological biofilm crucial for periodontal homeostasis [12].

Consequently, the critical interplay between periodontal microbial pathogens and persistent gingival inflammation constitutes the core of both the initiation and progression of PD [6].

Regarding the present study, after the thorough research of recent literature data, this is the first study, which quantifies the level of bacterial load, using qRT-PCR technology, for a set of 12 microbial strains with different periodontal pathogenicity (high, medium, and low) in two groups of Caucasian periodontal patients, namely young adults aged between 30 and 40 years, with or without associated MAL.

Referring to the MAL -PD binomial, the clinical study conducted by Batih et al. (2024) revealed that all 76 young patients with initial MAL, included in the study, developed PD, regardless of the bacterial load [32].

Byrne et al. (2009) reported that *Treponema denticola*, *Porphyromonas gingivalis*, and *Tannerella forsythia* are commonly found together in the subgingival plaque of patients with chronic PD [33,34]. In addition, Mohanty et al. (2019) points out that *Porphyromonas gingivalis* or *Treponema denticola* are rarely found in subgingival plaque unassociated with *Treponema forsythia* [35].

In accordance with the results of previous studies, our analysis of bacteria with a high degree of pathogenicity indicated the presence of the three bacterial species in both groups, specifying that the concentrations of the red complex bacteria, *Tannerella forsythia* (*p* = 0.020, mean = 6.04 ± 0.72 vs. mean = 4.4 ± 1.89), *Treponema denticola* (*p* = 0.023, median = 4.32, IQR = 2.76–5.53 vs. median = 1.93, IQR = 0–3.19), and *Porphyromonas gingivalis* (*p* = 0.002, median = 5.64, IQR = 4.94–5.98 vs. median = 2.48, IQR = 0–4.05) were significantly increased in patients with MAL compared to patients without MAL.

The higher concentration of pathogenic bacteria in patients with MAL and PD compared with those with PD alone may be explained by a combination of anatomical, functional, and microbiological factors.

Firstly, the accessibility and efficiency of oral hygiene are compromised in patients with MAL. MAL creates areas that are difficult to access for effective hygiene [36,37,38,39], making these areas ideal for plaque build-up due to compromised mechanical cleaning [40].

Secondly, food retention and plaque formation are exacerbated by malpositioned teeth. These teeth create interdental spaces where food can stagnate, providing a nutrient-rich substrate for bacteria [41], which facilitates the formation of plaque and biofilm, which are primary causes of PD [42,43].

Thirdly, patients with MAL experience alterations in salivary flow and its distribution in the oral cavity [44]. Saliva has antibacterial properties and helps to mechanically wash away food debris and bacteria. The uneven distribution of saliva can create oral microclimates that favor the colonization of pathogenic bacteria [45].

Lastly, inflammation and the local immune response are crucial factors in the disturbance of bacterial homeostasis in patients with MAL, since MALs can induce chronic inflammation through the constant irritation of the periodontal tissues [17,39,46]. Chronic inflammation can compromise the local immune response, facilitating the colonization and proliferation of pathogenic bacteria [47]. Moreover, inflammation can induce changes in the oral microflora, favoring the bacteria associated with PD [48].

Mechanical stress and the distribution of occlusal forces must also be considered, as MAL can cause the uneven distribution of occlusal forces, leading to mechanical stress on periodontal tissues [49]. This stress can cause microtrauma that favors bacterial infiltration and exacerbates the inflammatory response [50].

Additionally, MAL can influence the structure and composition of the bacterial biofilm, favoring the colonization of periodontal pathogens such as *Treponema denticola*, *Porphyromonas gingivalis*, and *Tannerella forsythia* [51]. These bacteria interact synergistically, increasing the pathogenicity of the biofilm and exacerbating periodontal inflammation [52,53].

Mohanty et al. (2019) observed that, in the subgingival plaque of patients with chronic periodontitis, the concentration of *Tannerella forsythia* exceeds that of *Porphyromonas gingivalis*, suggesting that *Tannerella forsythia* colonizes the plaque prior to *Porphyromonas gingivalis* and *Treponema denticola* [35]. Socransky et al. (1998) suggested a sequential colonization, where *Tannerella forsythia* may precede *Porphyromonas gingivalis*, thus establishing a foundational niche for subsequent pathogens [34]. Supporting this hypothesis, Popova et al. (2013) observed that disease-prone periodontal sites exhibited increased concentrations of *Tannerella forsythia* prior to the emergence of *Porphyromonas gingivalis*, reinforcing the concept of *Tannerella forsythia* as an early colonizer in these pathogenic successions [54].

Conversely, Kumar et al. (2003) provided a nuanced view, asserting that the presence of *Tannerella forsythia* does not consistently predict the arrival of *Porphyromonas gingivalis* and suggesting that microbial interactions are dynamic and influenced by various factors rather than following a linear progression [55]. Darveau (2009) emphasized the synergistic nature of interactions within the red complex, proposing that biofilm pathogenicity is more attributable to collective microbial interactions than to a sequential colonization process. This perspective argues against a simplistic linear model of pathogen succession, highlighting the complex interdependencies among these bacteria [56].

Griffen et al. (2012) provided compelling genomic evidence of co-occurrence patterns among *Tannerella forsythia*, *Porphyromonas gingivalis*, and *Treponema denticola*. Their research elucidates that, although certain preferential microbial partnerships may exist, the actual sequence of colonization is subject to considerable variation influenced by the host’s unique genetic and immunological environment [57].

Expanding on these findings, our study revealed a higher concentration of *Tannerella forsythia* compared to *Porphyromonas gingivalis* in both study groups. Notably, these concentrations were statistically elevated in patients with MAL compared to those without MAL (Table 2, Figure 1), suggesting that MAL create favorable conditions for the growth of periodontopathogenic bacteria, thereby exacerbating the progression of periodontal disease (PD).

The affinity of *Tannerella forsythia* for environments shaped by MAL is supported by the findings of Patano et al. (2023), who postulated that crowding significantly amplifies bacterial colonization by creating niches that are less amenable to routine hygienic interventions [58]. Chapple et al. (2018) highlighted how MAL create niches that foster the growth of specific pathogens, including *Tannerella forsythia*. This study elucidated that MAL complicate oral hygiene practices, thereby promoting plaque accumulation and bacterial colonization, exacerbating the microbial challenges associated with these MALs [59,60].

Further explorations of these dynamics are provided by Aas et al. (2005), who conducted a detailed microbial survey of complex oral biofilms. Their findings indicated that patients with MAL exhibited a significantly higher prevalence of periodontal pathogens such as *Tannerella forsythia*. This study suggests that MAL generate microenvironments which are particularly conducive to the survival and proliferation of bacteria associated with periodontal diseases, thus establishing a direct link between anatomical irregularities and microbial pathogenicity [60].

Building on this foundation, Kolenbrander et al. (2010) analyzed interbacterial interactions within plaque biofilms impacted by MAL. Their research demonstrated that dental misalignments significantly alter the microbial ecology of the oral cavity. They noted the enhanced co-aggregation and co-adhesion among bacteria, including *Tannerella forsythia*, which, when interacting synergistically with other periodontal pathogens, could substantially increase their pathogenic potential [61].

In addition to the “red complex” bacteria, *Aggregatibacter actinomycetemcomitans*, facultatively anaerobic coccobacillus belonging to the purple complex, is another significant periodontal pathogen often associated with more aggressive forms of PD [34]. In our study, *Aggregatibacter actinomycetemcomitans*, a bacterium with major pathogenic effects even at low concentrations, was identified only in patients without MAL. It is responsible for triggering gingival inflammatory phenomena and the destruction of supporting tissues. Its impact on PD involves the significant loss of alveolar bone and increased tooth mobility [62].

The exclusive identification of *Aggregatibacter actinomycetemcomitans* in patients with periodontitis elucidates the intricate interplay between MAL and microbial colonization in the oral cavity. This selective presence of the bacterium underscores the significant influence of anatomical features on the oral microbial ecosystem and serves as an essential focal point for scientific investigation.

*Aggregatibacter actinomycetemcomitans* is renowned for its virulence in aggressive forms of periodontitis, primarily due to its production of leukotoxin, which disrupts the host’s immune defense. This virulence factor is pivotal in the pathogenesis of PD, as emphasized by Höglund Åberg et al. (2014), who highlighted its critical role in promoting periodontal destruction [63].

The absence of *Aggregatibacter actinomycetemcomitans* in patients with MAL might reflect its ecological specificity. However, contrasting findings from Fine et al. (2019) and Nørskov-Lauritsen et al. (2019) indicate that *Aggregatibacter actinomycetemcomitans* was present in various cases regardless of MAL, which calls for further investigation to delineate the influence of structural variations on microbial behavior and PD pathogenesis [19,64,65].

This discrepancy highlights the complexity of microbial dynamics within the oral cavity and underscores the necessity for more nuanced research to unravel how MAL affects bacterial colonization and the consequent development of PD.

The study of bacteria with a medium degree of pathogenicity indicated that, among the orange complex bacteria, only the concentrations of *Eikenella corrodens* (*p* = 0.040, mean = 4.55 ± 1.02 vs. mean = 3.23 ± 1.56), *Campylobacter rectus* (*p* < 0.001, mean = 4.2 ± 0.56 vs. mean = 1.8 ± 1.51), and *Prevotella intermedia* (*p* = 0.043, median = 5.04, IQR = 0–5.49 vs. median = 0, IQR = 0–3.39)—all Gram-negative bacteria characterized by a moderate association with PD—were significantly increased in patients with MAL compared to those without MAL. In contrast, *Fusobacterium nucleatum*, a bacterium strongly associated with the development of chronic PD due to its properties of adhering to other periodontopathogenic microorganisms, and *Prevotella nigrescens*, a bacterium moderately associated with PD often identified alongside other pathogenic germs in active disease sites, were significantly increased in patients without MAL.

The observed differences in bacterial profiles between patients with MAL and those without, specifically in terms of the variations in the prevalence of certain orange complex bacteria, provide insightful clues into the complex interplay between dental anatomy and periodontal pathogen colonization. The significant increase in *Eikenella corrodens, Campylobacter rectus*, and *Prevotella intermedia* in patients with MAL may suggest that dental anomalies create favorable conditions for these bacteria, possibly due to altered niche environments that impede effective oral hygiene or alter the ecological balance favoring these specific organisms.

Conversely, the higher prevalence of *Fusobacterium nucleatum* and *Prevotella nigrescens* in patients without MAL could be indicative of a different microbial ecology that may still predispose to PD through other pathways [66,67]. *Fusobacterium nucleatum* is particularly noted for its ability to act as a bridge between early and late colonizers in the biofilm development process, enhancing the pathogenic potential of the dental plaque through co-aggregation with other pathogens [68,69]. Meanwhile, *Prevotella nigrescens* is often implicated in sites with active periodontal destruction due to its virulence factors and synergistic interactions with other pathogens [20,70].

The study of bacteria with a low degree of pathogenicity indicated that, among the green complex bacteria, only the concentrations of *Capnocytophaga sputigena* (*p* = 0.011, median = 5.91, IQR = 5.47–6.17 vs. median = 4.63, IQR = 3.83–5.64) and *Capnocytophaga gingivalis* (*p* = 0.007, median = 5.87, IQR = 5.34–6.03 vs. median = 4.4, IQR = 3.5–5.71), which are characterized by a minor association with PD, were significantly increased in patients with MAL compared to those without MAL. These species, which are part of the green complex typically associated with health, exhibit a minor association with PD but seem to proliferate differently in altered anatomical environments.

*Capnocytophaga sputigena* and *Capnocytophaga gingivalis* are part of the normal oral flora and are generally considered low-risk in terms of periodontal pathogenicity. However, their role in the oral microbiome might be more complex than previously understood [71,72,73,74].

In terms of oral health implications, the increased bacterial load in patients with MAL and PD leads to more intense inflammatory responses and the faster progression of periodontal tissue damage [39]. The higher concentrations of periodontal bacteria observed in patients with MAL primarily result from the anatomical and functional challenges posed by these abnormalities, which make effective oral hygiene difficult. This elevated bacterial presence exacerbates the inflammatory response, contributing to more severe PD. Addressing MAL through orthodontic or surgical interventions, coupled with thorough periodontal treatment, can effectively reduce bacterial burden and significantly improve overall oral health outcomes. Therefore, managing MAL comprehensively is crucial for mitigating periodontal complications and promoting lasting oral health.

## 5. Suggestions for Future Research

Reflecting on the insights of this study regarding the relationship between MAL and PD, we propose the following future research: (1) initiating longitudinal studies to track the progression of PD in patients with MAL, to understand temporal and causal relationships; (2) expanding the demographic scope of study populations to include a diverse ethnicities, ages, and socioeconomic backgrounds, thereby enhancing the generalizability of the findings; (3) applying advanced genomic sequencing to perform a comprehensive analysis of the oral microbiome, delineating a broader spectrum of bacterial species that impact periodontal health; (4) developing interdisciplinary clinical studies and evaluating the combined treatment approaches for managing PD in patients with MAL; and (5) undertaking mechanistic studies to explore the biological and biomechanical pathways by which MAL influences microbial colonization and PD pathogenesis, aiming to identify new therapeutic targets.

Through these focused research initiatives, our aim is to pave the way toward more sophisticated, precise, and personalized treatment paradigms that effectively address the unique challenges presented by MAL in the context of PD.

## 6. Limitations of the Study

This study has several methodological constraints that could impact its validity and broader applicability: (1) as a pilot study with a limited sample size focused on cooperative Caucasian patients aged 30–40, the findings might not be generalizable to other demographics; (2) examining only 12 specific periodontopathogenic bacteria may not fully capture the complexity of the oral microbiome in PD; (3) the use of qRT-PCR for bacterial quantification, while precise, does not provide insights into the active or viable bacterial community, limiting the understanding of microbial dynamics; (4) the cross-sectional design captures only a snapshot in time, restricting the ability to observe longitudinal changes or establish causality in disease progression.

These limitations necessitate the cautious interpretation of the results and underscore the importance of employing more inclusive and advanced methodologies in future research to fully explore the interactions between MAL, periodontal health, and bacterial load.

## 7. Conclusions

This study demonstrates a significant relationship between the presence of MAL and the increased bacterial load of specific periodontopathogenic bacteria, notably those belonging to the red and orange complexes.

Elevated concentrations of highly pathogenic bacteria, *Treponema denticola*, *Tannerella forsythia*, and *Porphyromonas gingivalis* were observed in patients with MAL compared to those without, suggesting that MAL creates favorable conditions for the growth of periodontopathogenic bacteria, thereby exacerbating the progression of PD.

Moreover, the study revealed increased levels of the moderate pathogenic bacteria, *Eikenella corrodens*, *Campylobacter rectus*, and *Prevotella intermedia* in patients with MAL versus those without, indicating a broad influence of anatomical abnormalities on the oral microbial profiles.

Additionally, higher concentrations of low-pathogenic bacteria, *Capnocytophaga sputigena* and *Capnocytophaga gingivalis*, were found in patients with MAL compared to those without.

This study highlights the significant impact that MAL has on the dynamics of the periodontal microbiota and underscores the necessity for an integrated interdisciplinary treatment approach that addresses both microbial imbalances and MAL to effectively manage PD. By implementing highly personalized and comprehensive therapeutic interventions, we can significantly improve the periodontal health of patients with MAL.

## Figures and Tables

**Table 1:** Characteristics of the analyzed patients.

Parameter	Total	No Malocclusions	With Malocclusions	*p*
N	20	10 (50%)	10 (50%)	
Age (mean ± SD)	37.35 ± 3.16	36 ± 3.97	38.7 ± 1.16	0.054 *
Sex (Nr., %)	14 (70%) Male	6 (60%) Male	8 (80%) Male	0.628 **
Education (Nr., %)				
ISCED 3-4	11 (55%)	7 (70%)	4 (40%)	0.370 **
Environment (Nr., %)				
Urban	11 (55%)	6 (60%)	5 (50%)	1.000 **
Smoking (Nr., %)				
Ex-smoker	4 (20%)	3 (30%)	1 (10%)	0.500 **
Smoker	5 (25%)	3 (30%)	2 (20%)	
Non-smoker	11 (55%)	4 (40%)	7 (70%)	
Periodontitis (Nr., %)				
Family history	11 (55%)	4 (40%)	7 (70%)	0.370 **
Periodontitis–Stage (Nr., %)				
Stage I (1–3 mm)	2 (10%)	2 (20%)	0 (0%)	0.554 **
Stage II (3–4 mm)	10 (50%)	4 (40%)	6 (60%)	
Stage III (4–6 mm)	8 (40%)	4 (40%)	4 (40%)	
Stage IV (>6 mm)	0 (0%)	0 (0%)	0 (0%)	
Periodontitis–Location (Nr., %)				
Generalized	17 (85%)	9 (90%)	8 (80%)	1.000 **
Localized	3 (15%)	1 (10%)	2 (20%)	
Malocclusions–Types (Nr., %)				
Crowding	9 (90%)	0 (0%)	9 (90%)	0.318 **
Open bite	1 (10%)	0 (0%)	1 (10%)	

* Student *t*-test, ** Fisher’s exact test, N = Number of patients, SD = standard deviation, ISCED = International Standard Classification of Education (ISCED).

## Data Availability

Data are contained within the article.
